# Salt‐tolerant native plants have greater responses to other environments when compared to salt‐tolerant invasive plants

**DOI:** 10.1002/ece3.5368

**Published:** 2019-06-19

**Authors:** Muxin Liu, Huixuan Liao, Shaolin Peng

**Affiliations:** ^1^ State Key Laboratory of Biocontrol School of Life Sciences Sun Yat‐sen University Guangzhou China

**Keywords:** combined stresses, invasion, mechanism, rapid evolution, salt stress, stress tolerance, trade‐off

## Abstract

1. The strong expansion potential of invasive plants is often attributed to fast adaptive responses to stress. However, the evolution of tolerance to one stressor may affect the responses to other stressors. Currently, it remains unclear what effect the evolution to one stressor might have on the responses to other single or combined stressors. Moreover, it is unknown how this might differ between invasive and native species.
2. Invasive plants (*Mikania micrantha* and *Bidens pilosa*) and native plants (*Merremia hederacea* and *Sida acuta*) from low‐ and high‐salinity habitats were grown under control and stressful conditions [salt stress, water stress (drought/waterlogging), and their combinations]. We explored the effects of evolved salt tolerance on the responses to water stress/combined stresses and the underlying trait mechanisms.
3. The high‐salinity populations of all species exhibited stronger salt tolerance than the low‐salinity populations. As to the tolerance to other stressors, the high‐salinity and low‐salinity populations of the invasive species were similar, whereas the high‐salinity populations of the native species exhibited stronger tolerance than the low‐salinity populations under most stress treatments. However, the enhanced salt tolerance in native species was accompanied by reduced total biomass under control condition. The stress tolerance of native species correlated with leaf production rate and allocation to root, while the performance of native species under control condition correlated with leaf morphology and carbon assimilation rate. This suggests a trade‐off between salt tolerance and performance in the native but not the invasive species, probably resulting from altered phenotypic/physiological traits.

**Synthesis:**

Our work suggests that the evolution of tolerance to one stressor may have stronger effects on the tolerance to other stressors of the native compared with the invasive species. This may be a new paradigm to explain the greater advantage of invasive vs. native species in highly stressful habitats.

## 1 INTRODUCTION

Stressful habitats were once believed to have strong resistance against exotic plant invasions (Baruch & Fernandez, 1993; Lugo, 1998), because stresses can act as natural barriers to unfit individuals (Alpert, Bone, & Holzapfel, 2000; Bockelmann & Neuhaus, 1999). However, the incidences of exotic plants invading some rarely inhabited, stressful habitats have increased dramatically (Caño, Mendizabal, Baquero, & Herrera, 2016). The greater expansion potential of invasive compared with native species is often attributed to fast adaptive responses to stresses (Liao, D'Antonio, Chen, Huang, & Peng, 2016). Plants might evolve a stronger tolerance to one stressor in order to persist in a particular habitat (Ahmad, Ashraf, & Ali, 2010), and such evolution has been proposed to incur costs on plant performance (Bijlsma & Loeschcke, 2005; Lachmuth, Durka, & Schurr, 2011) and affect the responses to other stressors (Brenes‐Arguedas, Roddy, & Kursar, 2013). So far, whether the evolution of stronger tolerance to one stressor may affect the invasive and native plants differently with respect to the costs on performance and their responses to other stressors has received little attention (but see Turner, Hufbauer, & Rieseberg, 2014). The differential effects of evolved stress tolerance on invasive and native species may be a new paradigm to understand the advantage of invasive species over native species.

The evolution of stronger stress tolerance can weaken plant performance under benign conditions, resulting in a trade‐off between performance and stress tolerance (Bijlsma & Loeschcke, 2005; Lachmuth et al., 2011). For instance, increased tolerance to flooding reduced the shoot biomass of *Centaurea diffusa* under benign condition (Turner et al., 2014). However, evidence also showed that the invasive populations of *C. diffusa* may be released from the trade‐off between plant performance and drought tolerance (Turner et al., 2014), which may explain its invasion success in North America. Moreover, the evolution of tolerance to one stressor can affect the tolerance to other stressors. For instance, evolution in response to salt or drought stress can provide “cross tolerance” to each other (Ashraf & O'leary, 1996), whereas evolutions in response to drought, waterlogging, or mechanical stresses may compromise shade tolerance (Huber, Brouwer, Wettberg, During, & Anten, 2013; Niinemets & Valladares, 2006).

The effect of the evolution of tolerance to one stressor on plant performance or plant tolerance to other stressors is dictated by functional traits. In general, a stressor can trigger trait responses in plants which are shaped by the interactions among a suite of physiological processes (Dolferus, 2014). Therefore, whenever the evolution of stronger tolerance to one condition results in trait variations that reduce plant fitness under other conditions, a cost occurs. Because such trait responses are stress‐specific (Dolferus, 2014), the evolution of tolerance to one stressor may enhance or weaken the tolerance/responsiveness to other stressors (Ashraf & O'leary, 1996; Eränen, Nilsen, Zverev, & Kozlov, 2009), depending on how the key traits respond to the focal stressors. It has been reported that decreased leaf stomatal density and faster stomatal closure in the drought‐tolerant ecotype may enhance its salt tolerance but weaken its heat tolerance because of the lower transpiration rate induced by drought (Chakraborty et al., 2018; Yang et al., 2015). Moreover, combined stressors can differ from its stress components by triggering more complicated interactions (Zandalinas, Rivero, Martínez, Gómez‐Cadenas, & Arbona, 2016; Zhang & Sonnewald, 2017). For example, plants may open their stomata under heat but close their stomata under salt/drought stress. However, plants open their stomata under combined heat and salt stresses (Mittler, 2006) but close their stomata under combined heat and drought stresses (Rizhsky, Liang, & Mittler, 2002; Rizhsky et al., 2004). Given the common co‐occurrence of multiple stressors in nature (Savvides, Ali, Tester, & Fotopoulos, 2016), it is necessary to explore how the evolution of tolerance to one stressor affects the tolerance to combined stressors.

The intertidal zone of Shenzhen Bay, Guangdong, used to be inhabited by a healthy mangrove forest. In 1998, however, a dam was constructed, which exposed a large patch of the soil of the mangrove forest, resulting in the rapid invasion of an invasive vine (*Mikania micrantha*) (Mao, Lai, Zhao, & Yang, 2011; Yu & Yang, 2011). During the past two decades, this area was subsequently colonized by several other species, including an invasive herb (*Bidens pilosa* var. *radiata*), a native vine (*Merremia hederacea*), and a native herb (*Sida acuta*). Evidently, high soil salinity combined with waterlogging inhibited the colonization of the above‐mentioned species into the mangrove forest, whereas high soil salinity alone failed to suppress the invaders. Genetic analyses have supported the strong capability of *M. micrantha* to adaptively evolve in response to salt stress (Guo et al., 2018; Wang, Chen, Zan, Wang, & Su, 2012). Hence, the incidence of the successful invasion of *M. micrantha* into Shenzhen Bay suggests that some populations may have rapidly evolved strong salt tolerance. Therefore, Shenzhen Bay provides an ideal research system to explore plant response to the combined salt and water stresses following the evolution of salt tolerance.

In the current study, we aim to explore what effect of the evolution to one stressor might have on the responses to other stressors and how this differs between invasive and native species. Using Shenzhen Bay as the study system, we specifically address the following questions: (a) Do high‐salinity environments enhance the salt tolerance of plant species? Does this tolerance differ between native and invasive species? (b) Does this tolerance come with consequences when in low‐salinity environments? Does this differ between native and invasive species? (c) Does this tolerance affect plant responses to other stressful environments? Does this differ between native and invasive species? (d) What are the underlying trait mechanisms for the salt tolerance and the potential trade‐offs between salt tolerance and plant responses to other conditions?

## 2 MATERIALS AND METHODS

### 2.1 Study species

We chose the four species mentioned in the Introduction as the study species, including an invasive vine, *M. micrantha*, an invasive herb, *B. pilosa*, a native vine, *M. hederacea,* and a native herb, *S. acuta*. Both chosen invasive species were Asteraceae because most invasive species in Guangdong Province belonged to Asteraceae family (Yue et al., 2011). *M. micrantha* and *B. pilosa* were first introduced into Hong Kong and became widespread in Southern China, where they have formed dense populations in various habitats (Xu et al., 2012). Both species are considered as the most noxious weeds in Guangdong Province (Yue et al., 2011). *M. hederacea* (Convolvulaceae) and *S. acuta* (Malvaceae) are common species in Southern China, which frequently co‐occur with *M. micrantha* and *B. pilosa* in field.

### 2.2 Seed collection

The high‐ and low‐salinity habitat were located in Shenzhen Bay, Guangdong Province, China (22.49–22.53°N, 113.96–114.01°E) and Xiaoguwei Island, Guangdong Province, China (23.03–23.07°N, 113.35–113.41°E), respectively. In Shenzhen Bay, there were a few remaining patches of mangrove forests heavily invaded by *M. micrantha* and *B. pilosa*, which were surrounded but not waterlogged by seawater (soil electrical conductivity = 6.46 ± 0.27 ms/cm; measured by an in situ salinometer [OK‐TY1, Oukeqi]). In Xiaoguwei Island, a large number of invasive species were introduced when the government started the construction of a university town in 2003 (soil electrical conductivity = 1.45 ± 0.24 ms/cm). Both habitats have been colonized by the four study species for at least 10 years and thus are suitable for our comparative study. During December 2014 and January 2015, we collected the seeds of each species from the two source populations (i.e., high‐ and low‐salinity populations). Seeds were collected from dense monocultures at the sites fully exposed to sunlight. Each population was represented by at least 60 maternal plants for herbs and 100 inflorescences for vines, which were collected from more than 6 sites to cover an area of at least 800 m^2^.

### 2.3 Greenhouse experiment

#### 2.3.1 Growth medium and seedling preparation

Each pot (21 cm in diameter and 18 cm in depth) was filled with 2.0 kg of a soil mixture consisting of 1:1 gardening soil and sand. The gardening soil was purchased from a commercial supplier (Jiffy Substrates), while the sand was obtained from a construction material market and sieved through a 2‐mm mesh.

About 500 seeds per population of each species were sown in seedling trays with a 1:1 gardening soil and sand mixture in a greenhouse at the east campus of Sun Yat‐Sen University on Xiaoguwei Island (23.08°N, 113.40°E). After 2 weeks since seed emergence, 36 seedlings of each species per seed origin with similar sizes were selected and transplanted into the pots prepared at the previous step. The initial stem length (SL*_i_*) and initial leaf number (LN*_i_*) of the main stems of all individuals were recorded. Six out of the 36 individuals per species from each habitat were randomly harvested and dried at 60°C for 72 hr to measure their initial weight. The other 30 individuals were subjected to stress treatments.

#### 2.3.2 Stress treatments

Six stress treatments were manipulated: (a) control (80% soil water mass [Mw]), (b) salt stress (80% Mw with NaCl concentration at 155 mmol/L), (c) drought stress (40% Mw), (d) waterlogging stress (160% Mw), (e) combined salt and drought stresses (40% Mw with NaCl concentration at 155 mmol/L level), and (f) combined salt and waterlogging stresses (160% Mw with NaCl concentration at 155 mmol/L level).

To manipulate soil water content, we first determined the average 100% soil water mass (Mw) per pot at the level of 100% field soil water capacity (FWC) (Hillel & van Bavel, 1976). Another three randomly selected pots of soil were dried in the sun and obtained the standard curve of FWC by regressing FWC against the readings of a portable soil moisture meter (ZD‐06, ZD Instrument Co.). To manipulate drought and control condition, we calculated the reading of the soil moisture meter at 40% FWC (drought condition) and 80% FWC (control condition) (Hsiao, 1973), which were 30% and 78% soil moisture, respectively. To manipulate waterlogging conditions, we measured the depth of the water in pots that is required to achieve an FWC of 160%, which was 17.5 cm (2.4 cm above the soil surface). During the whole experiment, the soil moisture of the drought and control condition was maintained at 30% and 78%, respectively, while the water depth in the pots under the waterlogging treatment was maintained at 17.5 cm.

For the salt stress, we maintained the concentration of NaCl at about 150 mmol/L by dissolving 5.4 g, 2.7 g, and 10.8 g NaCl with 30 ml distilled water and adding the resulted solutions to the pots assigned to salt, combined salt and drought and combined salt and waterlogging treatments, respectively. This NaCl concentration for salt stress treatment was selected to mimic moderate soil salinity level in field (i.e., 100–200 mmol/L) (Barrett‐Lennard, Bennett, & Colmer, 2008). During the whole experiment, the electrical conductivity of the pots under salt stress and combined salt and water stresses was constantly maintained at around 10 ms/cm, which was 8 times greater than the electrical conductivity of the pots under control and single water stress (Table S1).

The positions of all pots were randomized every week. All plants were harvested before flowering. Because of the shorter life span of herbs compared with vines, the treatments on the pair of herbs lasted for 4 weeks from April to May, while the treatments with the pair of vines lasted for 16 weeks from late April to late August 2015. During the experiment, all plants were subjected to full‐light condition with a temperature range between 20°C and 30°C.

### 2.4 Trait measurement

After harvest, all individual plants were separated into leaves (i.e., six fully expanded leaves on the main stems and the remaining leaves), stems, and roots. All plant parts were washed free of soil before being dried at 60°C for 72 hr and weighed. Nine leaf‐, growth‐, and allocation‐related traits were measured, including the relative growth rate of total biomass (RGR_TM_), stem length (RGR_SL_), and leaf number (RGR_LN_) as well as the specific leaf area (SLA), leaf weight ratio (LWR), stem weight ratio (SWR), root weight ratio (RWR), leaf area ratio (LAR), and net assimilation rate (NAR). The measurement and calculation of each trait value are shown in Table 1.

**Table 1:** The list of studied leaf‐, growth‐, and allocation‐related traits

Trait (Abbreviation)	Equation	Unit	Notes
Relative growth rate of total biomass (RGR_TM_)	RGR_TM_ = (lnTM*_f_* − lnTM*_i_*)/*t*	g g^−1^ day^−1^	TM, SL, and LN are total biomass, stem length, and leaf number, respectively. *t* is the length of the experimental period. *f* denotes final value, while *i* denotes initial value. LN*_i_* and SL*_i_* were measured before the experimental treatments. TM*_i_* was calculated by the dry weight of six randomly harvested individuals (averaging the weight for vines; using initial plant height as a covariate for the regression of the weight for herbs)
Relative growth rate of stem length (RGR_SL_)	RGRSL = (lnSL*_f_* − lnSL*_i_*)/*t*	cm cm^−1^ day^−1^	
Relative growth rate of leaf number (RGR_LN_)	RGRLN = (lnLN*_f_* − lnLN*_i_*)/*t*	pc pc^−1^ day^−1^	
Specific leaf area (SLA)	SLA = leaf area/leaf mass	cm^2^/g	SLA was measured using six fully expanded leaves on the main stem. Leaf area was measured using a LI‐3100C Area Meter (LI‐COR) prior to oven‐drying the leaves for weighing
Leaf weight ratio (LWR)	LWR = total leaf mass/total biomass	g/g	
Stem weight ratio (SWR)	LWR = total stem mass/total biomass	g/g	
Root weight ratio (RWR)	LWR = total root mass/total biomass	g/g	
Leaf area ratio (LAR)	LAR = SLA × LWR	cm^2^/g	
Net assimilation rate (NAR)	NAR = RGR_TM_/LWR	g cm^−2^ day^−1^	

### 2.5 Statistical analyses

All statistical analyses were conducted using SPSS 23.0 (version 23.0; IBM SPSS Statistics). In our study, due to the complexity of stress combinations, we treated each stress combination as an integrated stressor (see Mittler, 2006), instead of treating it as the interactive result of different stressors. Hence, the single stress and the combined stresses were treated equally in our statistical analysis, so that we will be able to see the effect of salt/water stress individually or jointly without worrying that their effects will be masked by each other.

#### 2.5.1 Quantifying stress tolerance

To quantify stress tolerance, we calculated the percent difference in total biomass of the individuals treated by stress and control condition:

(1) $$T=\left(Ms-Mc\right)/Mc\times 100\%$$

where *T* is the tolerance. *M* is the average total biomass. s and c denote stress treatment and control condition, respectively.

#### 2.5.2 Evolution of salt tolerance

To test the evolution of salt tolerance, we performed a two‐way ANOVA to compare the total biomass of the populations from high‐ and low‐salinity habitats of each species in response to salt stress. In this model, salt treatment (control vs. salt stress), habitat (high‐ vs. low‐salinity habitat), and their interaction were selected as the independent variables, whereas total biomass was selected as the dependent variable. A significant interaction between salt treatment and habitat indicates a significant difference in the salt tolerance of high‐ and low‐salinity populations.

#### 2.5.3 Salt tolerance and low‐salinity environments

To test the effect of evolved salt tolerance on plant performance in low‐salinity environments, we carried out a one‐way ANOVA using habitat (high‐ vs. low‐salinity habitat) as an independent variable and total biomass under control condition as the dependent variable. Each study species was analyzed separately. *t* Test was adopted to compare the difference in the total biomass between high‐ and low‐salinity populations for the following trait analysis.

In addition, we explored the correlation between the change in salt tolerance and the change in the total biomass under control condition for each species to determine whether there is a trade‐off between salt tolerance and performance.

#### 2.5.4 Salt tolerance and other stressors

To test the effect of different stress treatment on plant performance, we performed one‐way ANOVA using treatment (control, salt, drought, waterlogging, combined salt and drought, and combined salt and waterlogging) as the independent variable and total biomass as the dependent variable. Each population (high‐salinity or low‐salinity population) of each study species was tested separately. The difference in plant performance among treatments was tested using Tukey HSD post hoc analysis.

To test the effects of evolved salt tolerance on the responses to water stress and combined salt and water stresses, we replaced salt treatment with one of the other four stress treatments (i.e., drought/waterlogging/combined salt and drought/combined salt and waterlogging) one at a time in the above‐mentioned model and reran the test. A significant interaction between treatment and habitat indicates the occurrence of salt‐induced changes in the tolerance/responsiveness to other stressors. Because the final biomass of the two herbs, *B. pilosa* and *S. acuta*, significantly correlated with the initial stem length (the Pearson correlation coefficients for these two species were 0.258 [*p* = 0.047] and 0.534 [*p* < 0.001], respectively), we additionally included initial stem length as a covariate in the ANOVA models for these two species.

#### 2.5.5 Underlying trait mechanisms

To explore the underlying trait mechanisms for the differential responses, we looked at the potential cost/benefit of certain trait evolution on plant performance under control condition and plant tolerance to different stressors.

To serve this purpose, we first tested whether there were significant trait differences between the high‐ and low‐salinity populations of each species under each treatment by conducting two‐sample *t* tests.

Then, we calculated the percent trait, biomass, and tolerance differences between the high‐ and low‐salinity populations using the following equation:

(2) $$D_{\text{trait}}=\left({\text{Trait}}_{h}-{\text{Trait}}_{l}\right)/\left|{\text{Trait}}_{l}\right|\times 100\%,$$

(3) $$D_{\text{mass}}=\left({\text{Mass}}_{h}-{\text{Mass}}_{l}\right){\text{/Mass}}_{l}\times 100\%,$$

(4) $$D_{\text{tolerance}}=\left(T_{h}-T_{l}\right)/\left|T_{l}\right|\times 100\%,$$

where *D*_trait_, *D*_mass_, and *D*_tolerance_ are the percent difference of trait, total biomass, and tolerance, respectively; *h* and *l* refer to the values of the high‐ and low‐salinity populations of each species, respectively.

To visualize the correlation between trait evolution and plant performance/stress tolerance, we drew a heat map of the trait difference between the high‐ and low‐salinity populations for the four study species under each treatment, with reference to Hodgins et al. (2015) and Shaar‐Moshe, Blumwald, and Peleg (2017). As a result, six heat maps corresponding to six treatments were produced. For each treatment, if the pattern of the variations of a trait parallels the pattern of the performance/tolerance of the four study species, then there is a positive correlation between the changes in the trait and plant performance/tolerance. Likewise, if the pattern for a trait is opposite to the pattern of plant performance/tolerance, there is a negative trait–performance/tolerance correlation.

## 3 RESULTS

### 3.1 Evolution of salt tolerance

**Figure 1:**
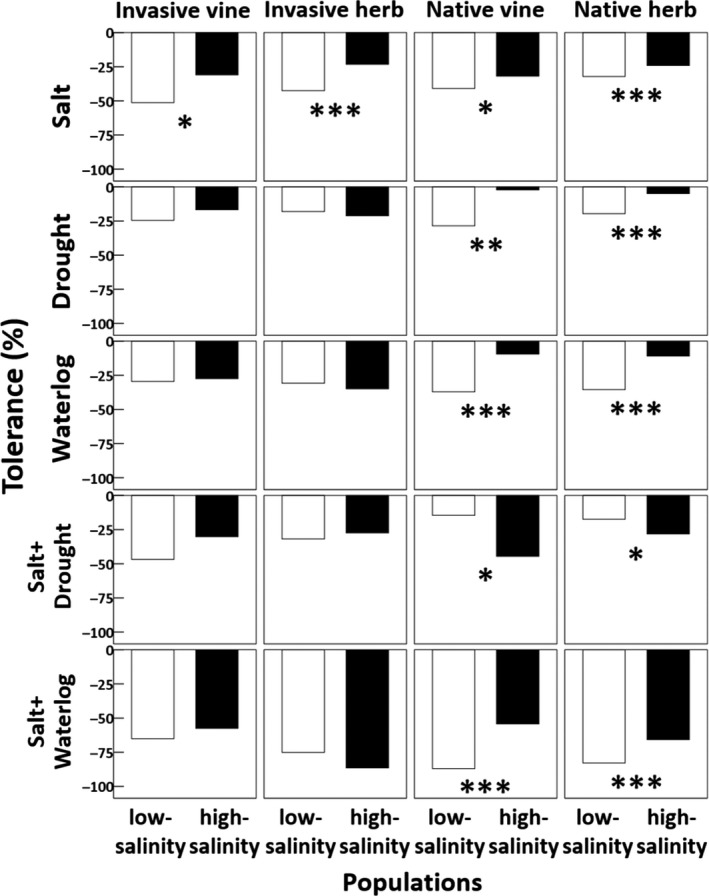
Tolerance of the low‐ and high‐salinity populations of each species to different stressors. Tolerance was calculated as the percent difference in total biomass of the individuals treated by stress and control. The significance of tolerance difference between the two populations was determined by the significance of habitat‐stress treatment interaction. Asterisks indicate significant differences (*, *p* ≤ 0.05; **, *p* ≤ 0.01; and ***, *p* ≤ 0.001). Refer to statistics in Table 2 and Table S2

There was a significant interactive effect between habitat and salt treatment on the total biomass of every species (Table 2), indicating that the high‐salinity populations had stronger salt tolerance compared with the low‐salinity populations across all species (Figure 1).

**Table 2:** ANOVA/ANCOVA outputs for individual and interactive effects of habitat (H) and stress treatments (salt [S]/drought [D]/waterlogging [W]/combined salt and drought [S + D]/combined salt and waterlogging [S + W]) on total biomass

Source	*df*	Invasive vine		Invasive herb		Native vine		Native herb	
		*F*	*p*	*F*	*p*	*F*	*p*	*F*	*p*
SL*_i_*	1	/	/	**65.32**	**<0.001**	/	/	**530.91**	**<0.001**
H	1	**29.71**	**<0.001**	**18.59**	**0.001**	**37.36**	**<0.001**	**194.37**	**<0.001**
S	1	**155.08**	**<0.001**	**395.18**	**<0.001**	**76.09**	**<0.001**	**379.70**	**<0.001**
H * S	1	**6.30**	**0.023**	**41.34**	**<0.001**	**7.03**	**0.017**	**34.31**	**<0.001**
SL*_i_*	1	/	/	**53.28**	**<0.001**	/	/	**392.99**	**<0.001**
H	1	**14.27**	**0.002**	**12.38**	**0.003**	**21.22**	**<0.001**	**147.06**	**<0.001**
D	1	**38.10**	**<0.001**	**206.17**	**<0.001**	**14.74**	**0.001**	**69.30**	**<0.001**
H * D	1	0.77	0.394	0.37	0.551	**11.94**	**0.003**	**19.65**	**<0.001**
SL*_i_*	1	/	/	**105.72**	**<0.001**	/	/	**695.83**	**<0.001**
H	1	**11.51**	**0.004**	**13.06**	**0.003**	**19.15**	**<0.001**	**108.92**	**<0.001**
W	1	**107.75**	**<0.001**	**803.79**	**<0.001**	**34.74**	**<0.001**	**444.73**	**<0.001**
H * W	1	0.01	0.912	0.46	0.51	**17.15**	**0.001**	**191.97**	**<0.001**
SL*_i_*	1	/	/	**62.01**	**<0.001**	/	/	**740.68**	**<0.001**
H	1	**27.29**	**<0.001**	0.09	0.771	**129.76**	**<0.001**	**652.22**	**<0.001**
S + D	1	**154.41**	**<0.001**	**332.14**	**<0.001**	**42.10**	**<0.001**	**281.70**	**<0.001**
H * S + D	1	4.39	0.052	3.38	0.086	**5.34**	**0.034**	**4.83**	**0.044**
SL*_i_*	1	/	/	**36.28**	**<0.001**	/	/	**392.06**	**<0.001**
H	1	**16.60**	**0.001**	**17.54**	**0.001**	3.12	0.099	**36.67**	**<0.001**
S + W	1	**571.94**	**<0.001**	**2,776.48**	**<0.001**	**278.03**	**<0.001**	**2,492.74**	**<0.001**
H * S + W	1	0.10	0.751	2.17	0.161	**44.75**	**<0.001**	**176.61**	**<0.001**

Note

Initial stem length (SL*_i_*) was used as a covariate for the invasive herb and the native herb. Significant results are shown in bold.

### 3.2 Salt tolerance and low‐salinity environments

Under control condition, the total biomass of the high‐salinity populations did not differ from the low‐salinity populations for the invasive vine (*p* = 0.074) or the invasive herb (*p* = 0.083; Table S3). For the native species, however, the high‐salinity populations had smaller total biomass compared with the low‐salinity populations for both the vine (*p* = 0.001) and the herb (*p* = 0.046; Table S3). Because the high‐salinity populations of all species exhibited stronger salt tolerance compared with the low‐salinity populations, the above results suggest a trade‐off between salt tolerance and performance under control condition in the native but not the invasive species.

### 3.3 Salt tolerance and other stressors

For the stress treatments other than salt, there were significant interactive effects between habitat and stress treatment on the total biomass of the native species but not the invasive species (Table 2). Specifically, the high‐salinity populations of the native species exhibited stronger tolerance to drought/waterlogging/combined salt and waterlogging but weaker tolerance to combined salt and drought compared with the low‐salinity populations (Figure 1; Table 2). In contrast, the high‐salinity populations of the invasive species did not differ from the low‐salinity populations with respect to the tolerance to water stress and combined salt and water stresses (Figure 1; Table 2).

**Figure 2:**
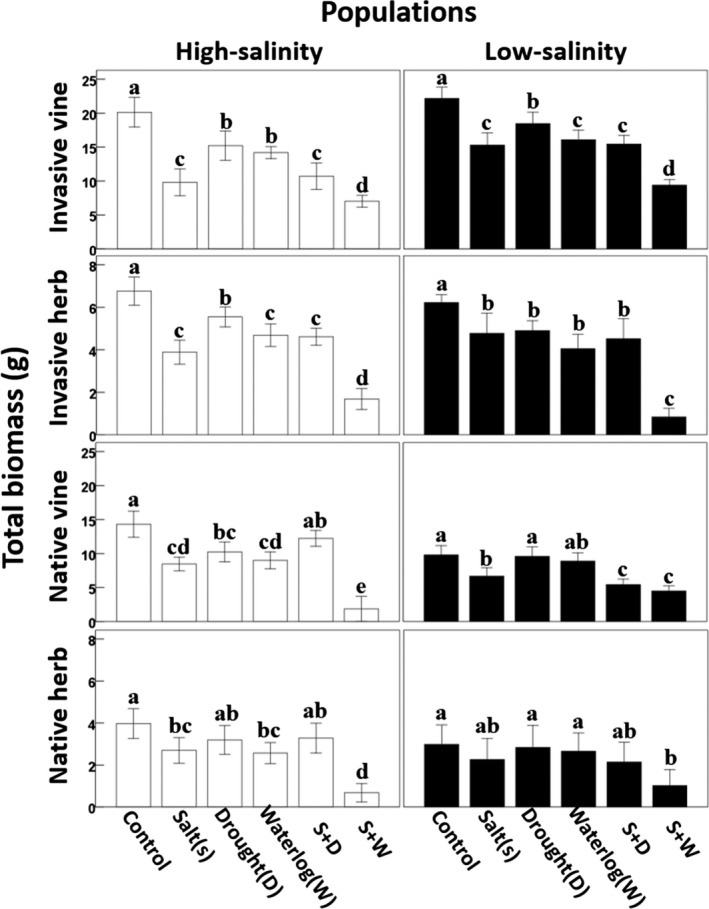
The responsiveness of the low‐ and high‐salinity populations of each species to different stressors. Different lowercase letters indicate significant differences

Both the high‐salinity and low‐salinity populations were significantly affected by stress treatments for the invasive vine and herb, indicating that the high‐salinity populations of the invasive species were equally responsive to different stress treatments as the low‐salinity populations (Figure 2; Table 3). In contrast, the high‐salinity populations were less affected by the stress treatments comparing with the low‐salinity populations for the native vine and herb, indicating that adaptation to high salinity may reduce the responsiveness of native species to other stressors (Figure 2; Table 3).

**Table 3:** ANOVA/ANCOVA outputs for the overall effect of stress treatments on the total biomass of the low‐ and high‐salinity populations

Population	Source	*df*	Invasive vine		Invasive herb		Native vine		Native herb	
			*F*	*p*	*F*	*p*	*F*	*p*	*F*	*p*
Low‐salinity	SL*_i_*	1	/	/	87.25	**<0.001**	/	/	441.89	**<0.001**
	Treatment	5	53.53	**<0.001**	81.49	**<0.001**	55.39	**<0.001**	25.43	**<0.001**
High‐salinity	SL*_i_*	1	/	**/**	93.89	0.900	/	/	742.99	**<0.001**
	Treatment	5	61.78	**<0.001**	54.12	**<0.001**	29.00	**<0.001**	4.51	**0.005**

Note

Initial stem length (SL*_i_*) was used as a covariate for the invasive herb and the native herb. Significant results are shown in bold.

### 3.4 Underlying trait mechanisms

**Figure 3:**
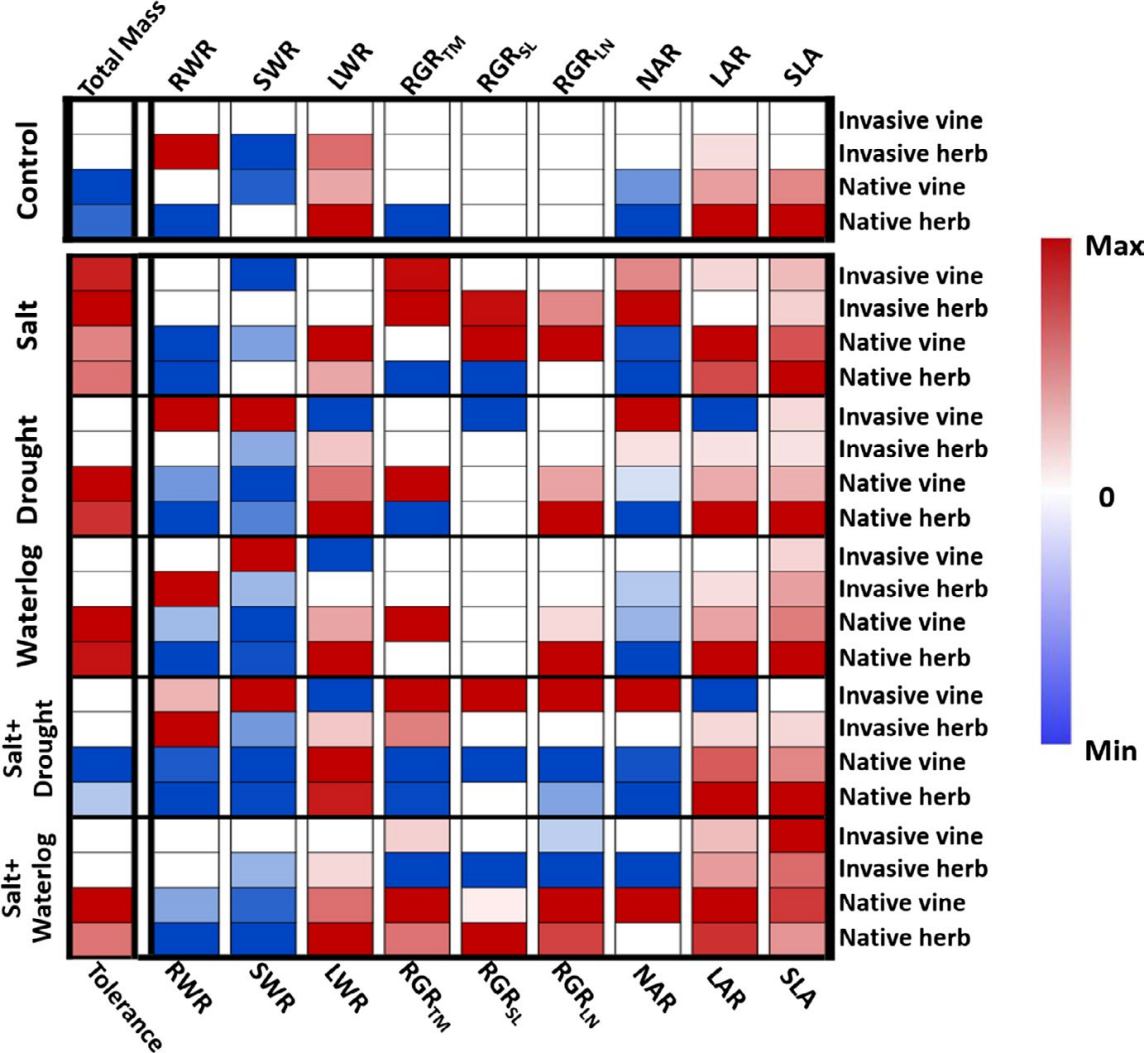
The traits difference between high‐ and low‐salinity populations basing on *t* tests. For each cell, red, white, and blue squares, respectively, indicate positive, 0, and negative difference between the two populations, quantified by the percent difference in the trait values between the high‐ and low‐salinity populations. LAR, leaf area ratio; LWR, leaf weight ratio; NAR, net assimilation rate; RGR_LN_, relative growth rate of leaf number; RGR_SL_, relative growth rate of stem length; RGR_TM_, relative growth rate of total biomass; RWR, root weight ratio; SLA, specific leaf area; SWR, stem weight ratio. See trait descriptions in Table 1. Refer to statistics in Table S4

By looking at the traits corresponded with salt tolerance, we found that the high‐salinity populations of all study species had larger SLA under salt treatment compared with the low‐salinity populations (Figure 3). The *t* values for the invasive vine, invasive herb, native vine, and native herb were −3.11 (*p* = 0.014), −3.54 (*p* = 0.008), −26.35 (*p* < 0.001) and −12.01 (*p* < 0.001), respectively. This suggests that larger SLA may enhance salt tolerance.

As to the traits corresponded with plant performance under control condition, we found that the high‐salinity populations of the native species consistently had larger SLA (for both vine and herb: *p* < 0.001) and smaller NAR (for both vine and herb: *p* < 0.001) compared with the low‐salinity populations under control treatment (Figure 3; Table S4). In contrast, such patterns were absent in the invasive species (Figure 3; Table S4). This indicates that the worse performance of the high‐ versus low‐salinity population of the native species under control condition may be due to the increased SLA and decreased NAR.

As to the traits corresponded with the difference in the tolerance to water stress, we found that the high‐salinity populations of the native species consistently had larger relative leaf production rate (RGR_LN_) than the low‐salinity population under drought treatment (for the vine: *p* = 0.028; for the herb: *p* < 0.001) and waterlogging treatment (for the vine: *p* = 0.019; for the herb: *p* < 0.001; Figure 3; Table S4). In contrast, no such patterns were found in the invasive species (Figure 3; Table S4). This indicates that the stronger tolerance to water stress of the high‐ versus low‐salinity populations of the native species may correspond with increased RGR_LN_ under drought/waterlogging treatment.

As to combined salt and drought, no trait variation seemed to account for the weaker tolerance of the high‐ versus low‐salinity populations of the native species. As to combined salt and waterlogging, we found that the high‐salinity populations of the native species consistently had smaller RWR than the low‐salinity populations (for the vine: *p* = 0.048; for the herb: *p* < 0.001; Figure 3; Table S4). There was a trend of reduced RWR in the high‐ versus low‐salinity population of the invasive herb (*p* = 0.051), and no such pattern was found in the invasive vine (Figure 3; Table S4). This indicates that the stronger tolerance to combined salt and waterlogging of the high‐ versus low‐salinity population may correspond with decreased RWR.

## 4 DISCUSSION

Invasive plants have been proven to be capable of rapid evolution in response to stresses, which profoundly contribute to their strong expansion potential (Kollmann & Bañuelos, 2004; Liao, Gurgel, Pal, Hooper, & Callaway, 2016; Poll, Naylor, Alexander, Edwards, & Dietz, 2009). However, how the rapid evolution of tolerance to one stressor may further affect the expansion potential of invasive plants through affecting the responses to other stressful environments has not received much attention. Our study is among the first to explore the effect of evolved tolerance to one stressor on the responses to combined stressors and how this may differ between invasive and native species.

### 4.1 Salt tolerance and performance under low‐salinity environments

According to our results, we found enhanced salt tolerance in the high‐salinity populations of all study species. However, such evolution has incurred a cost in the native species under control condition (Figures 1 and 2). The trade‐off between plant performance and stress tolerance has also been reported in previous studies (Hodgins & Rieseberg, 2011; Lachmuth et al., 2011). In our study, this trade‐off was only found in the native but not the invasive species, suggesting that the invaders may be released from this trade‐off. Similarly, Turner et al. (2014) had also reported a trade‐off between plant performance and drought tolerance in the native but not the invasive populations of *C. diffusa*. Hence, we suggest that the lower cost incurred by the evolved stress tolerance in the invasive versus native species may be a general mechanism for explaining the greater success of invasive versus native species in various stressful habitats.

### 4.2 Salt tolerance and responses to other stressors

It has been commonly reported that the evolution of tolerance to one stressor may strengthen, weaken, or has little effect on plant responses to other stressors (Ashraf & O'leary, 1996; Huber et al., 2013; Niinemets & Valladares, 2006). In the current study, we found that the evolution of salt tolerance had prominent effects on the tolerance to other stressors of the native but not of the invasive species. Combined with the results of weakened performance of native species under control condition, we suggest that stronger salt tolerance had reduced the responsiveness of native species to other stressors in most cases, while having little effect of the responsiveness of invasive species (Figure 2).

### 4.3 Underlying trait mechanisms

According to our results, the species‐specific responses may result from the distinct trait evolution induced by salt stress.

We found that the high‐salinity populations had larger SLA compared with the low‐salinity populations under salt stress. Larger SLA was also found in salt‐tolerant cultivars under salt treatment (Praxedes, Lacerda, DaMatta, Prisco, & Gomes, 2010). It may explain the enhanced salt tolerance of the high‐salinity populations by increasing light/carbon capture, which partly compensates the biomass loss under salt stress (Godoy, Valladares, & Castro‐Díez, 2011; Poorter & Garnier, 2007). Meanwhile, larger SLA in the high‐ versus low‐salinity populations under control condition was exclusively observed in the native species, which seemed to hinder plant performance when environments are favorable (Figures 2 and 3). Thus, our results suggest that larger SLA, which often corresponds with rapid leaf turnover rate and more intense self‐shading (Grotkopp, Rejmánek, & Rost, 2002), may be maladapted under favorable conditions. Our results also suggest the co‐occurrence of enhanced salt tolerance and decreased NAR under control condition in the native species. Net assimilation rate is often found to positively associate with the maximum photosynthetic rate (Li, Schmid, Wang, & Paine, 2016). Thus, salt‐induced decrease in NAR may explain the weakened performance of the native species under control conditions.

In addition, the reduced responsiveness to water stress of native species was found to be accompanied by increased leaf production rate, which supports the previous notion that faster leaf production may enhance plant performance under stressful conditions by increasing photosynthetic carbon gain (Meng et al., 2013). The reduced responsiveness to combined salt and waterlogging may be partially explained by reduced allocation to root. This supports the idea that less allocation to root may enhance plant tolerance to combined salt and waterlogging stresses because a relatively larger proportion of shoot biomass benefits from greater water and nutrient uptake per root biomass (Rubio, Oesterheld, Alvarez, & Lavado, 1997; Ye, Tam, Wong, & Lu, 2003). It is still not clear why different traits predominated the adaptive responses to different stress combinations.

### 4.4 The potential advantages of invasive versus native species

Due to human activities, the co‐occurrence of salt and water stresses has become increasingly common (Dale, Jager, Wolfe, & Efroymson, 2018), and they pose severe threats to natural ecosystems (Setter & Waters, 2003; Touhami et al., 2015). Rapid evolution has been identified as an important mechanism that facilitates exotic plant invasion (Molina‐Montenegro et al., 2018; Poll et al., 2009). Despite extensive studies on plants responses to single stress, experimental studies that focused on the responses to combined stresses are very limited (reviewed by Mahalingam, 2015), especially those concerning invasive species (Stoler, Sudol, Mruzek, & Relyea, 2018). Our study proved that some invasive plants may gain greater advantages over native species following the evolution of stronger salt tolerance and thus have greater potential to invade highly stressful habitats.

## 5 CONCLUSION

Our results suggest that the evolution of stronger salt tolerance had incurred great costs in the performance of the native species under control condition and weakened their responsiveness to other stressors except for combined salt and drought stresses. The release from the trade‐off between performance and stress tolerance may enable the invasive species to pre‐adapt to other stressful conditions, which may be a new paradigm to explain the advantage of invasive over native species.

## CONFLICT OF INTEREST

None declared.

## AUTHORS' CONTRIBUTIONS

M.L. and S.P. conceived the ideas and designed the study. M.L. conducted field surveys, performed the experiments, and analyzed the data. M.L. and H.L. led the writing of the article. H.L. and S.P. revised the article. All authors contributed substantially to the drafts and gave final approval for publication.

## Data Availability

The data associated with this publication are deposited at Dryad data repository. Provisional https://doi.org/10.5061/dryad.ss5gp34. Data files title: Total biomass and trait values of the low‐ and high‐salinity populations (invasive/native & vine/herb) in response to single/combined stresses.
