# Supplementary material for: Salt‐tolerant native plants have greater responses to other environments when compared to salt‐tolerant invasive plants
Source: Ecol Evol. 2019 Jun 19;9(13):7808–18. doi: 10.1002/ece3.5368 (PMC6635938; doi:10.1002/ece3.5368)
Supplement: Supplementary file 1 [file ECE3-9-7808-s001.docx]

**Supporting Information**

**Table S1. The mean electrical conductivity of the soils under different treatments.** The electrical conductivity of each treatment was measured by an in-situ salinometer (OK-TY1, Oukeqi, China). Mean ± S.E. are shown. Different letters indicate statistical differences detected by One-way ANNOVA with Turkey’s test in post hoc (P<0.05).

| Treatments | Control | Drought | Waterlogging | Salt | Salt+Drought | Salt+Waterlogging |
| --- | --- | --- | --- | --- | --- | --- |
| EC (ms/cm) | 1.14 ± 0.07 a | 1.19 ± 0.04 a | 1.20± 0.05 a | 10.75± 0.09 b | 10.86 ± 0.10 b | 10.81 ± 0.10 b |

**Table S2.** **The tolerance to different stress treatments in low- and high-salinity populations of each species.** The significant differences in the stress tolerance between the two populations are shown in bold (refer to the effects of habitat and stress treatment in Table 2).

| **Stress** | **Invasive vine** | |  | **Invasive herb** | |  | **Native vine** | |  | **Native herb** | |
| --- | --- | --- | --- | --- | --- | --- | --- | --- | --- | --- | --- |
|  | **Low** | **High** |  | **Low** | **High** |  | **Low** | **High** |  | **Low** | **High** |
| **Salt (S)** | **51.3%** | **31.0%** |  | **42.5%** | **23.3%** |  | **40.9%** | **31.9%** |  | **32.1%** | **24.1%** |
| **Drought (D)** | 24.5% | 16.7% |  | 18.0% | 21.2% |  | **28.5%** | **2.2%** |  | **19.7%** | **4.9%** |
| **Waterlogging (W)** | 29.5% | 27.4% |  | 30.7% | 34.9% |  | **37.1%** | **9.5%** |  | **35.5%** | **10.9%** |
| **S+D** | 46.8% | 30.2% |  | 31.8% | 27.4% |  | **14.5%** | **44.6%** |  | **17.4%** | **28.1%** |
| **S+W** | 65.1% | 57.6% |  | 75.1% | 86.5% |  | **87.0%** | **54.3%** |  | **82.9%** | **65.8%** |

**Table S3. The performance of the low- and high-salinity populations of each species under control condition.**

|  | **df** | **Low-salinity populations** | **High-salinity populations** | **t** | **P** |
| --- | --- | --- | --- | --- | --- |
| **Invasive vine** | 8 | 20.14 | 22.17 | -2.05 | 0.074 |
| **Invasive herb** | 8 | 6.76 | 6.23 | 1.98 | 0.083 |
| **Native vine** | 8 | 14.31 | 9.81 | **5.31** | **0.001** |
| **Native herb** | 8 | 3.97 | 2.98 | **2.36** | **0.046** |

Significant results are in bold.

**Table S4. The trait differences (TD) between the high- and low-salinity populations of each species under each treatment.** t values and degree of freedom (df) are shown. Significant results are shown in bold and marked by asterisks (*, P<0.05; **, P<0.01; ***, P<0.001). For performance (total biomass)/tolerance, the significance of percentage difference was referred to the t test/AN(C)OVA model in the maintext. SLA-specific leaf area; LAR-leaf area ratio; NAR-net assimilation rate; RGR_LN_-relative growth rate of leaf number; RGR_SL_-relative growth rate of stem length; RGRTM-relative growth rate of total biomass; LWR-leaf weight ratio; SWR-stem weight ratio; RWR-root weight ratio; TM-total biomass.

| **Treatment** | **Species** | **SLA** | | | **LAR** | | | **NAR** | | | **RGR_LN_** | | | **RGR_SL_** | | |
| --- | --- | --- | --- | --- | --- | --- | --- | --- | --- | --- | --- | --- | --- | --- | --- | --- |
|  |  | **df** | **t** | **TD** | **df** | **t** | **TD** | **df** | **t** | **TD** | **df** | **t** | **TD** | **df** | **t** | **TD** |
| **C** | **M.m** | 8.00 | 0.69 | -0.035 | 8.00 | 0.71 | -0.038 | 8.00 | -0.18 | 0.013 | 8.00 | -0.33 | 0.019 | 8.00 | 1.84 | -0.076 |
|  | **B.p** | 8.00 | -0.08 | 0.003 | **8.00** | **-5.24***** | **0.208** | 8.00 | 1.93 | -0.097 | 8.00 | -0.71 | 0.112 | 8.00 | -0.69 | 0.107 |
|  | **M.h** | **8.00** | **-15.93***** | **0.411** | **8.00** | **-13.91***** | **0.586** | **8.00** | **7.33***** | **-0.393** | 8.00 | 0.84 | -0.077 | 8.00 | 1.50 | -0.128 |
|  | **S.a** | **8.00** | **-29.58***** | **0.872** | **8.00** | **-23.57***** | **1.532** | **8.00** | **20.83***** | **-0.683** | 8.00 | 0.04 | -0.003 | 8.00 | -0.49 | 0.028 |
| **S** | **M.m** | **8.00** | **-3.11*** | **0.157** | **4.38** | **-3.27*** | **0.214** | **8.00** | **-2.61*** | **0.157** | 8.00 | 0.50 | -0.034 | 8.00 | 0.65 | -0.060 |
|  | **B.p** | **8.00** | **-3.54**** | **0.109** | 8.00 | -1.82 | 0.062 | **8.00** | **-8.71***** | **0.333** | **8.00** | **-2.55*** | **0.956** | **8.00** | **-3.48**** | **0.457** |
|  | **M.h** | **8.00** | **-26.35***** | **0.395** | **8.00** | **-25.51***** | **1.365** | **8.00** | **10.66***** | **-0.538** | **8.00** | **-6.92***** | **2.037** | **8.00** | **-2.46*** | **0.481** |
|  | **S.a** | **8.00** | **-12.01***** | **0.582** | **5.36** | **-12.81***** | **0.973** | **8.00** | **14.38***** | **-0.567** | 8.00 | 0.73 | -0.062 | **8.00** | **3.22*** | **-0.167** |
| **D** | **M.m** | **8.00** | **-3.86**** | **0.183** | **8.00** | **3.84**** | **-0.214** | 4.89 | -3.75 | 0.355 | 8.00 | -0.92 | 0.067 | **8.00** | **2.54*** | **-0.124** |
|  | **B.p** | **8.00** | **-4.52**** | **0.145** | **8.00** | **-7.26***** | **0.344** | **8.00** | **9.95***** | **-0.208** | 8.00 | -0.43 | 0.116 | 8.00 | -0.32 | 0.037 |
|  | **M.h** | **8.00** | **-14.51***** | **0.382** | **8.00** | **-27.74***** | **0.972** | **8.00** | **8.01***** | **-0.369** | **8.00** | **-2.69*** | **0.483** | 5.64 | -1.21 | 0.107 |
|  | **S.a** | **8.00** | **-30.74***** | **1.222** | **4.67** | **-23.83***** | **2.926** | **4.15** | **13.40***** | **-0.781** | **8.00** | **-10.95***** | **1.338** | 8.00 | -1.14 | 0.080 |
| **W** | **M.m** | **8.00** | **-3.85**** | **0.132** | 8.00 | 1.07 | -0.079 | 8.00 | -1.30 | 0.087 | 8.00 | -1.50 | 0.184 | 8.00 | -1.21 | 0.077 |
|  | **B.p** | **8.00** | **-9.55***** | **0.288** | **8.00** | **-6.21***** | **0.310** | **8.00** | **5.76***** | **-0.197** | 8.00 | -0.25 | 0.030 | 8.00 | 1.21 | -0.141 |
|  | **M.h** | **8.00** | **-11.83***** | **0.395** | **8.00** | **-12.71***** | **0.817** | **8.00** | **6.31***** | **-0.267** | **5.05** | **-3.38*** | **0.689** | 8.00 | -2.08 | 0.202 |
|  | **S.a** | **8.00** | **-20.52***** | **0.772** | **5.06** | **-21.10***** | **2.262** | **4.02** | **10.78***** | **-0.673** | **8.00** | **-10.52***** | **4.529** | 8.00 | -1.25 | 0.092 |
| **S+D** | **M.m** | 8.00 | -0.90 | 0.030 | **8.00** | **2.77*** | **-0.194** | **8.00** | **-4.08**** | **0.609** | **8.00** | **-10.59***** | **16.384** | **8.00** | **-3.26*** | **0.162** |
|  | **B.p** | **8.00** | **-4.57**** | **0.137** | **8.00** | **-4.21**** | **0.254** | 5.41 | 1.38 | -0.097 | 8.00 | -0.12 | 0.044 | 8.00 | -0.17 | 0.019 |
|  | **M.h** | **8.00** | **-13.27***** | **0.407** | **8.00** | **-20.33***** | **1.060** | **8.00** | **10.38***** | **-0.705** | **8.00** | **4.98***** | **-0.407** | **8.00** | **4.88***** | **-0.411** |
|  | **S.a** | **8.00** | **-21.69***** | **0.864** | **4.99** | **-19.18***** | **1.643** | **4.36** | **13.61***** | **-0.764** | **8.00** | **4.46**** | **-0.199** | 8.00 | 2.08 | -0.142 |
| **S+W** | **M.m** | **8.00** | **-7.06***** | **0.328** | **8.00** | **-2.77*** | **0.251** | 8.00 | -0.83 | 0.096 | **8.00** | **2.89*** | **-0.544** | 8.00 | 0.72 | -0.062 |
|  | **B.p** | **8.00** | **-6.20***** | **0.191** | **8.00** | **-6.92***** | **0.376** | **8.00** | **3.43**** | **-4.595** | **8.00** | **4.05**** | **-2.119** | **4.66** | **5.38***** | **-1.734** |
|  | **M.h** | **6.00** | **-6.50***** | **0.255** | **6.00** | **-9.77***** | **0.961** | **2.13** | **-5.16*** | **1.450** | **6.00** | **-5.37**** | **1.508** | **6.00** | **-7.12***** | **1.531** |
|  | **S.a** | **6.00** | **-4.35**** | **0.137** | **6.00** | **-8.77***** | **0.780** | 6.00 | -2.33 | 1.004 | **4.65** | **-7.46***** | **1.122** | **8.00** | **-2.61*** | **87.139** |

| **Treatment** | **Species** | **RGR_TM_** | | | **LWR** | | | **SWR** | | | **RWR** | | | **TM** | | |
| --- | --- | --- | --- | --- | --- | --- | --- | --- | --- | --- | --- | --- | --- | --- | --- | --- |
|  |  | **df** | **t** | **TD** | **df** | **t** | **TD** | **df** | **t** | **TD** | **df** | **t** | **TD** | **df** | **t** | **TD** |
| **C** | **M.m** | 8.00 | 0.69 | -0.021 | 8.00 | 0.15 | -0.003 | 8.00 | 1.41 | -0.053 | 8.00 | -1.63 | 0.041 | 1.00 | 4.21 | 0.058 |
|  | **B.p** | 6.21 | -1.72 | 0.086 | **8.00** | **-4.60**** | **0.205** | **8.00** | **5.90***** | **-0.149** | **8.00** | **-4.19**** | **0.310** | 1.00 | 3.59 | 0.346 |
|  | **M.h** | 8.00 | 0.77 | -0.038 | **8.00** | **-5.81***** | **0.124** | **8.00** | **3.27**** | **-0.128** | 8.00 | 1.53 | -0.107 | **1.00** | **28.20***** | **-0.125** |
|  | **S.a** | **8.00** | **3.45**** | **-0.198** | **8.00** | **-8.05***** | **0.355** | 8.00 | -1.01 | 0.039 | **5.77** | **22.05***** | **-0.470** | **1.00** | **259.40***** | **-0.552** |
| **S** | **M.m** | **8.00** | **-3.85**** | **0.398** | 8.00 | -1.54 | 0.055 | **8.00** | **2.71*** | **-0.180** | 8.00 | -1.39 | 0.051 | **1.00** | **32.62***** | **0.069** |
|  | **B.p** | **8.00** | **-10.72***** | **0.412** | 8.00 | 2.01 | -0.043 | 8.00 | -0.74 | 0.016 | 8.00 | -1.29 | 0.145 | **1.00** | **60.69***** | **0.154** |
|  | **M.h** | 8.00 | -1.02 | 0.094 | **8.00** | **-14.24***** | **0.695** | **8.00** | **2.59*** | **-0.091** | **8.00** | **9.11***** | **-0.364** | **1.00** | **9.39*** | **-0.456** |
|  | **S.a** | **8.00** | **2.67*** | **-0.148** | **8.00** | **-5.82***** | **0.244** | 8.00 | 1.63 | -0.060 | **8.00** | **13.10***** | **-0.363** | **1.00** | **25.43***** | **-0.417** |
| **D** | **M.m** | 8.00 | -1.10 | 0.053 | **8.00** | **6.89***** | **-0.333** | **4.49** | **-6.23**** | **0.809** | **8.00** | **-2.86*** | **0.079** | **1.00** | **10.84*** | **0.109** |
|  | **B.p** | 8.00 | -1.27 | 0.066 | **8.00** | **-5.11***** | **0.174** | **5.04** | **3.99**** | **-0.076** | 8.00 | 0.61 | -0.062 | **1.00** | **8.84*** | **-0.068** |
|  | **M.h** | **8.00** | **-3.57**** | **0.241** | **8.00** | **-11.54***** | **0.426** | **8.00** | **5.49***** | **-0.169** | **8.00** | **8.13***** | **-0.317** | 1.00 | 0.78 | -0.386 |
|  | **S.a** | **8.00** | **3.02*** | **-0.138** | **8.00** | **-18.73***** | **0.766** | **5.31** | **4.79**** | **-0.114** | **8.00** | **18.04***** | **-0.575** | **1.00** | **20.84**** | **-0.651** |
| **W** | **M.m** | 8.00 | 0.14 | -0.005 | **5.66** | **3.29*** | **-0.187** | **5.38** | **-4.06**** | **0.530** | 8.00 | 1.18 | -0.052 | **1.00** | **9.98*** | **-0.067** |
|  | **B.p** | 8.00 | -1.30 | 0.053 | 8.00 | -0.57 | 0.018 | **8.00** | **3.01*** | **-0.077** | **8.00** | **-5.49***** | **0.846** | **1.00** | **17.21**** | **0.921** |
|  | **M.h** | **8.00** | **-4.39**** | **0.328** | **8.00** | **-6.08***** | **0.303** | **8.00** | **6.34***** | **-0.204** | **8.00** | **4.36**** | **-0.193** | 1.00 | 0.04 | -0.226 |
|  | **S.a** | 8.00 | -1.05 | 0.073 | **8.00** | **-20.12***** | **0.841** | **8.00** | **8.46***** | **-0.190** | **8.00** | **16.77***** | **-0.526** | **1.00** | **7.04*** | **-0.608** |
| **S+D** | **M.m** | **8.00** | **-3.36**** | **0.272** | **8.00** | **3.60**** | **-0.220** | **8.00** | **-2.59*** | **0.287** | **8.00** | **-3.90**** | **0.136** | **1.00** | **32.02***** | **0.192** |
|  | **B.p** | **8.00** | **-2.78*** | **0.137** | **8.00** | **-2.49*** | **0.103** | **8.00** | **4.73***** | **-0.096** | **8.00** | **-3.81**** | **0.460** | 1.00 | 1.28 | 0.500 |
|  | **M.h** | **8.00** | **7.74***** | **-0.390** | **8.00** | **-12.72***** | **0.466** | **8.00** | **9.61***** | **-0.176** | **8.00** | **8.56***** | **-0.323** | **1.00** | **174.50***** | **-0.384** |
|  | **S.a** | **8.00** | **6.41***** | **-0.379** | **8.00** | **-10.78***** | **0.418** | **8.00** | **5.76***** | **-0.172** | **8.00** | **13.96***** | **-0.367** | **1.00** | **355.00***** | **-0.425** |
| **S+W** | **M.m** | **8.00** | **-2.97*** | **0.342** | 8.00 | 1.31 | -0.060 | 8.00 | -1.84 | 0.177 | 8.00 | 1.43 | -0.105 | **1.00** | **29.55***** | **-0.135** |
|  | **B.p** | **8.00** | **3.49**** | **-5.624** | **8.00** | **-3.25*** | **0.157** | **8.00** | **3.86**** | **-0.136** | 8.00 | -2.29 | 0.297 | **1.00** | **14.87**** | **0.326** |
|  | **M.h** | **2.22** | **-4.71*** | **1.831** | **6.00** | **-7.76***** | **0.571** | **6.00** | **10.64***** | **-0.277** | **6.00** | **2.48*** | **-0.210** | **1.00** | **29.37**** | **-0.239** |
|  | **S.a** | **8.00** | **-2.50*** | **1.010** | **4.97** | **-5.20**** | **1.008** | **4.41** | **3.44*** | **-0.336** | **8.00** | **11.16***** | **-0.442** | **1.00** | **24.15**** | **-0.504** |
